# Exploratory analysis of immunochemotherapy compared to chemotherapy after EGFR‐TKI in non–small cell lung cancer patients with EGFR mutation: A multicenter retrospective study

**DOI:** 10.1111/1759-7714.14836

**Published:** 2023-03-03

**Authors:** Tae Hata, Chikara Sakaguchi, Keita Hirano, Hiroshi Kobe, Masaki Ishida, Takayuki Nakano, Yusuke Tachibana, Nobuyo Tamiya, Shinsuke Shiotsu, Takayuki Takeda, Tadaaki Yamada, Toshihide Yokoyama, Michiko Tsuchiya, Yukio Nagasaka

**Affiliations:** ^1^ Department of Respiratory Medicine Rakuwakai Otowa Hospital Kyoto Japan; ^2^ Department of Medical Oncology Rakuwakai Otowa Hospital Kyoto Japan; ^3^ Department of Nephrology Kyoto University Graduate School of Medicine Kyoto Japan; ^4^ Department of Respiratory Medicine Ohara Healthcare Foundation, Kurashiki Central Hospital Okayama Japan; ^5^ Department of Respiratory Medicine Graduate School of Medical Science, Kyoto Prefectural University of Medicine Kyoto Japan; ^6^ Department of Respiratory Medicine Japanese Red Cross Kyoto Daini Hospital Kyoto Japan; ^7^ Department of Respiratory Medicine Japanese Red Cross Kyoto Daiichi Hospital Kyoto Japan

**Keywords:** advanced non–small cell lung cancer, *EGFR* mutation, immunochemotherapy, immunotherapy, platinum‐based chemotherapy

## Abstract

**Background:**

Patients with epidermal growth factor receptor (*EGFR*)‐mutated, advanced non–small cell lung cancer have received immunochemotherapy as one of the treatment options after tyrosine kinase inhibitor (TKI) failure.

**Methods:**

We retrospectively examined *EGFR*‐mutant patients treated with atezolizumab‐bevacizumab‐carboplatin‐paclitaxel (ABCP) therapy or platinum‐based chemotherapy (Chemo) after EGFR‐TKI therapy at five institutions in Japan.

**Results:**

A total of 57 patients with *EGFR* mutation were analyzed. The median progression‐free survival (PFS) and overall survival (OS) in the ABCP (*n* = 20) and Chemo (*n* = 37) were 5.6 and 20.9 months, 5.4 and 22.1 months, respectively (PFS, *p* = 0.39; OS, *p* = 0.61). In programmed death‐ligand 1 (PD‐L1)–positive patients, median PFS in the ABCP group was longer than in the Chemo group (6.9 vs. 4.7 months, *p* = 0.89). In PD‐L1–negative patients, median PFS in the ABCP group was significantly shorter than in the Chemo group (4.6 vs. 8.7 months, *p* = 0.04). There was no difference in median PFS between the ABCP and Chemo groups in the subgroups of brain metastases, *EGFR* mutation status, or chemotherapy regimens, respectively.

**Conclusion:**

The effect of ABCP therapy and chemotherapy was comparable in *EGFR*‐mutant patients in a real‐world setting. The indication for immunochemotherapy should be carefully considered, especially in PD‐L1–negative patients.

## Graphical abstract

In non–small cell lung cancer patients with epidermal growth factor receptor (EGFR) mutations, the efficacy of atezolizumab‐bevacizumab‐carboplatin‐paclitaxel (ABCP) therapy and platinum‐based chemotherapy after EGFR‐tyrosine kinase inhibitor was found to be equivalent in real‐world setting. In programmed death‐ligand 1‐negative patients, the median progression‐free survival in the ABCP group was significantly shorter than in the chemotherapy group.

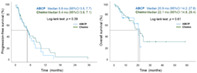

## INTRODUCTION

The therapeutic strategy of advanced lung cancer has entered a new phase with the advent of immunotherapy.^1^ Notably, for metastatic non–small cell lung cancer (NSCLC), front‐line treatment, the combination of immunotherapy and chemotherapy, is becoming the standard therapeutic strategy.^2^ The addition of immune checkpoint inhibitors (ICIs), such as programmed death 1 inhibitors, to platinum‐doublet chemotherapy has shown substantial survival benefits, even with low programmed death‐ligand 1 (PD‐L1) expression.^3^

For patients with epidermal growth factor receptor (*EGFR*) mutation, tyrosine kinase inhibitors (TKIs) are the primary treatment. Particularly, it has been suggested that using osimertinib as a first‐line treatment contributes greatly to improved prognosis.^4^, ^5^ In addition, post‐TKI therapy has become the focus and treatment strategies incorporating ICIs are being explored.

In recent years, a new treatment that uses ICIs effectively has been developed for *EGFR*‐mutant patients. In *EGFR*‐positive subgroup of the IMpower150 trial, atezolizumab‐bevacizumab‐carboplatin‐paclitaxel (ABCP) therapy (*n* = 34) had longer progression‐free survival (PFS) and overall survival (OS) compared to bevacizumab‐carboplatin‐paclitaxel (BCP) (*n* = 45) therapy: the median PFS and OS were 10.2 and 26.1 months and 6.9 and 20.3 months in ABCP and BCP therapies, respectively.^6^, ^7^ ICI combination chemotherapy other than atezolizumab has not been reported to be as effective as ABCP therapy in either prospective or retrospective studies,^8^, ^9^ and the combination of ICI and bevacizumab is reported to provide a clinical benefit.^6^, ^7^ However, in IMpower150 trial only one patient in the ABCP arm was treated with osimertinib, the standard of care, and some patients were treated with first‐line treatment without EGFR‐TKI therapy. Preclinical and clinical data have shown that EGFR‐TKI treatment diminishes the antitumor effects of immunity and that osimertinib reduces PD‐L1 expression.^10^, ^11^, ^12^ It is important to verify the effectiveness of ABCP treatment in real‐world setting and to examine the factors that influence treatment efficacy.

Our study aimed to compare the effect of ABCP therapy with chemotherapy in a real‐world setting and to identify the optimal post‐TKI treatment for *EGFR*‐mutant patients.

## MATERIALS AND METHODS

### Patients

We enrolled patients with *EGFR* mutated advanced (stage IIIB to IV) or recurrent NSCLC who were treated with ABCP therapy or platinum‐based chemotherapy (Chemo) between May 2016 and November 2021 at five participating institutions (Rakuwakai Otowa Hospital, Kurashiki Central Hospital, Kyoto Prefectural University of Medicine, Japanese Red Cross Kyoto Daini Hospital, and Japanese Red Cross Kyoto Daiichi Hospital) in Japan. The following patients were included: patients with histological or cytological diagnosis of NSCLC, patients with stage III/IV or postoperative recurrence who are not eligible for radical radiotherapy or surgery, patients who are determined to be *EGFR*‐positive by genetic testing, patients up to the second‐line treatment except for EGFR‐TKI, patients who received prior TKI treatment and with progressive disease. The patients with poorly controlled brain metastases were excluded. We retrospectively reviewed the patients' medical records and extracted clinical data. Clinical data was anonymized before sharing for analysis. This study was conducted according to the protocols approved by the institutional clinical research ethic committee of each participating hospital (No. 02–21‐00027).

### Study design

Patient clinical characteristics at the time of NSCLC diagnosis and initiation of ABCP therapy or chemotherapy were obtained from the medical records and included the following: sex, age, smoking history, comorbidity, PD‐L1 expression, histology, *EGFR* mutation status, Eastern Cooperative Oncology Group performance status (PS) at initiation of treatment, presence of pleural effusion, brain and liver metastases, treatment lines, types of prior EGFR‐TKI therapy, previous radiotherapy, previous pneumonectomy, clinical response to each treatment, and adverse events including immune‐related adverse event (irAE) type.

The primary objective was to assess PFS and OS between ABCP therapy and platinum‐based chemotherapy in *EGFR*‐mutant patients. The secondary objective was to evaluate objective response rate (ORR), disease control rate (DCR), and PFS according to PD‐L1 status, PFS according to *EGFR* mutation subgroup, PFS in patients with baseline brain metastases, and PFS according to chemotherapy regimens between ABCP therapy and platinum‐based chemotherapy.

PD‐L1 expression was examined before first‐line treatment or at the re‐biopsy after EGFR‐TKI failure using the PD‐L1 22C3 pharmDx (Dako). PD‐L1–positivity was defined as membranous staining ≥1% of the tumor cells. The secondary T790M mutation was not investigated.

### Treatments

Since the presentation of immunochemotherapy in the Japanese lung cancer guidelines in 2018 and the publication of the IMpower150 trial for *EGFR*‐mutant patients in 2019, ABCP therapy is now available for patients with good PS and an indication for bevacizumab (e.g., no bleeding lesions, no cavitary lesions, and no squamous cell cancer). Patients treated with ABCP therapy received four cycles every 21 days, followed by maintenance therapy. The doses were as follows: paclitaxel, 175 mg/m^2^; carboplatin, area under the concentration–time curve of 5 or 6 mg/mL per min; bevacizumab, 15 mg/kg; and atezolizumab, 1200 mg. Patients who were administered platinum‐based chemotherapy were treated every 21 days for up to four cycles, after which the patients who were treated with pemetrexed (PEM), bevacizumab, or both were transferred to maintenance therapy. The Chemo group includes following four groups: cisplatin‐based chemotherapy (with bevacizumab) (*n* = 6), cisplatin‐based chemotherapy (without bevacizumab) (*n* = 6), carboplatin‐based chemotherapy (with bevacizumab) (*n* = 3), carboplatin‐based chemotherapy (without bevacizumab) (*n* = 22) (Table S1). All therapy was discontinued at the time of unmanageable toxicity or disease progression.

The treatment responses were evaluated by a clinical physician of each institution according to the Response Evaluation Criteria in Solid Tumors version 1.1. Treatment efficacy was assessed by imaging evaluation every two cycles. The ORR was calculated by the rate of complete response (CR) and partial response (PR), and DCR was calculated by the rate of CR, PR, and stable disease. Treatment‐related adverse events were evaluated using the Common Terminology Criteria for Adverse Events version 5.0.

### Statistical analyses

The categorical variables are presented as counts and percentages. χ^2^ test was used to compare categorical variables, and *t*‐test was used to assess differences in continuous variables between the two groups. PFS was defined as the time from the onset of each treatment (ABCP therapy or platinum‐based chemotherapy) to progression or death. OS was defined as the time from the onset of each treatment to death because of any cause. PFS and OS were estimated using the Kaplan–Meier method, and the differences among the subgroups were compared using the log‐rank test. Two‐tailed *p* values <0.05 were considered statistically significant. We performed statistical analyses using IBM SPSS Statistics version 28 (IBM).

## RESULTS

### Patient characteristics

The median duration of the follow‐ups was 24.6 (range, 4.4–65.1) months. Of the 57 *EGFR‐*mutant patients, 20 and 37 received ABCP therapy and platinum‐based chemotherapy, respectively.

The median age was 70 (range, 43–80) years. The histology of all patients was adenocarcinoma. PD‐L1–positive tumors were identified in 22 (39%) of the 57 patients: ABCP (*n* = 10) and Chemo (*n* = 12). Osimertinib pretreatment was administered to 75% and 40.5% of the patients in the ABCP and Chemo groups, respectively; 11 (55%) and 13 (35%) patients in the first line. The baseline characteristics of the patients are summarized in Table 1.

**TABLE 1:** Characteristics of patients with *EGFR* mutation.

Characteristics	ABCP (*n* = 20), No. (%)	Chemo (*n* = 37), No. (%)	*p* value
Median age (range), years	63 (49–74)	73 (43–80)	<0.05
Male	10 (50)	21 (56.8)	0.63
ECOG performance status			0.12
0–1	19 (95)	36 (97.3)	
2	1 (5)	1 (2.7)	
Smoking history			0.21
Never	11 (55)	14 (37.8)	
Former/current	9 (45)	23 (62.2)	
Metastases			
Brain	10 (50)	10 (27)	0.08
Liver	1 (5)	0	0.17
EGFR mutation status			0.8
Exon 19 deletion	11 (55)	22 (59.5)	
Exon 21 L858R	8 (40)	12 (32.4)	
Others	1 (5)	3 (8.1)	
Treatment line			<0.05
Second line	11 (55)	33 (89.2)	
Third line	8 (40)	4 (10.8)	
Fourth or fifth line	1 (5)	0	
PD‐L1 TPS			0.42
≥1%	10 (50)	12 (32.4)	
<1%	6 (30)	16 (43.2)	
Unknown	4 (20)	9 (24.3)	
Prior osimertinib treatment	15 (75)	15 (40.5)	<0.05

Abbreviations: ABCP, atezolizumab‐bevacizumab‐carboplatin‐paclitaxel; Chemo, chemotherapy; ECOG, Eastern Cooperative Oncology Group; EGFR, epidermal growth factor receptor; PD‐L1, programmed death‐ligand 1; TPS, tumor proportion score.

In the Chemo group, 11 (30%) patients received cisplatin, 26 (70%) patients received carboplatin, and 10 (27%) patients received bevacizumab.

### Response rates

**FIGURE 1:**
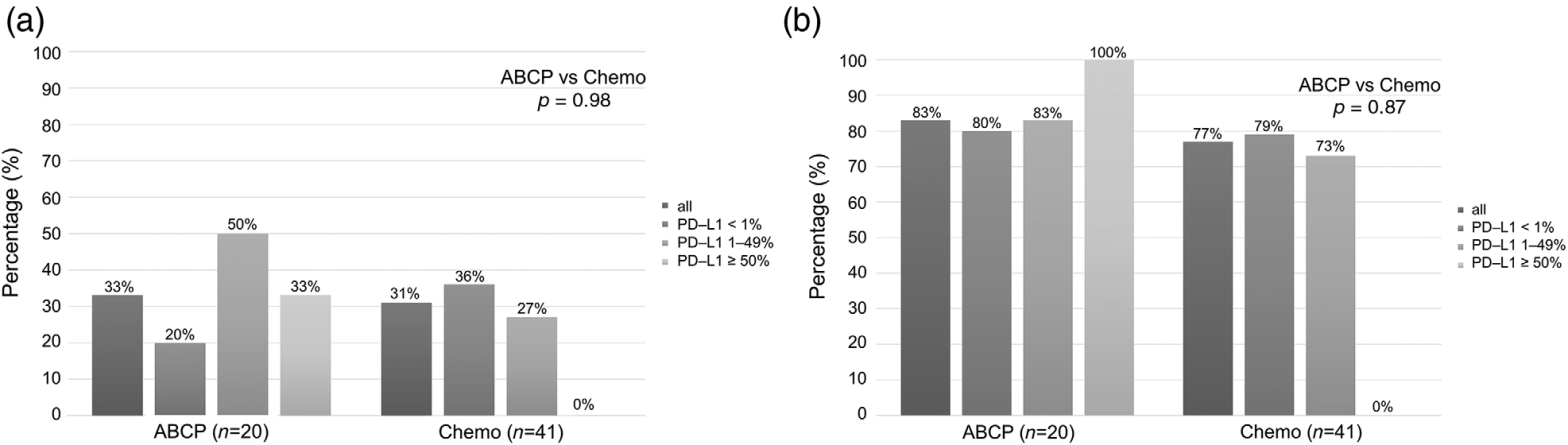
(a) Objective response rate in *EGFR*‐mutant patients. (b) Disease control rate in *EGFR*‐mutant patients: ABCP, atezolizumab‐bevacizumab‐carboplatin‐paclitaxel; Chemo, chemotherapy; EGFR, epidermal growth factor receptor; PD‐L1, programmed death‐ligand 1.

The ORRs in the ABCP and Chemo were 33% and 31%, respectively (*p* = 0.98). The DCRs were 83% and 77% in the ABCP and Chemo groups, respectively, (*p* = 0.87) (Figure 1). Of note, the ABCP group had higher DCR for all PD‐L1 expression.

### Progression‐free survival and overall survival

**FIGURE 2:**
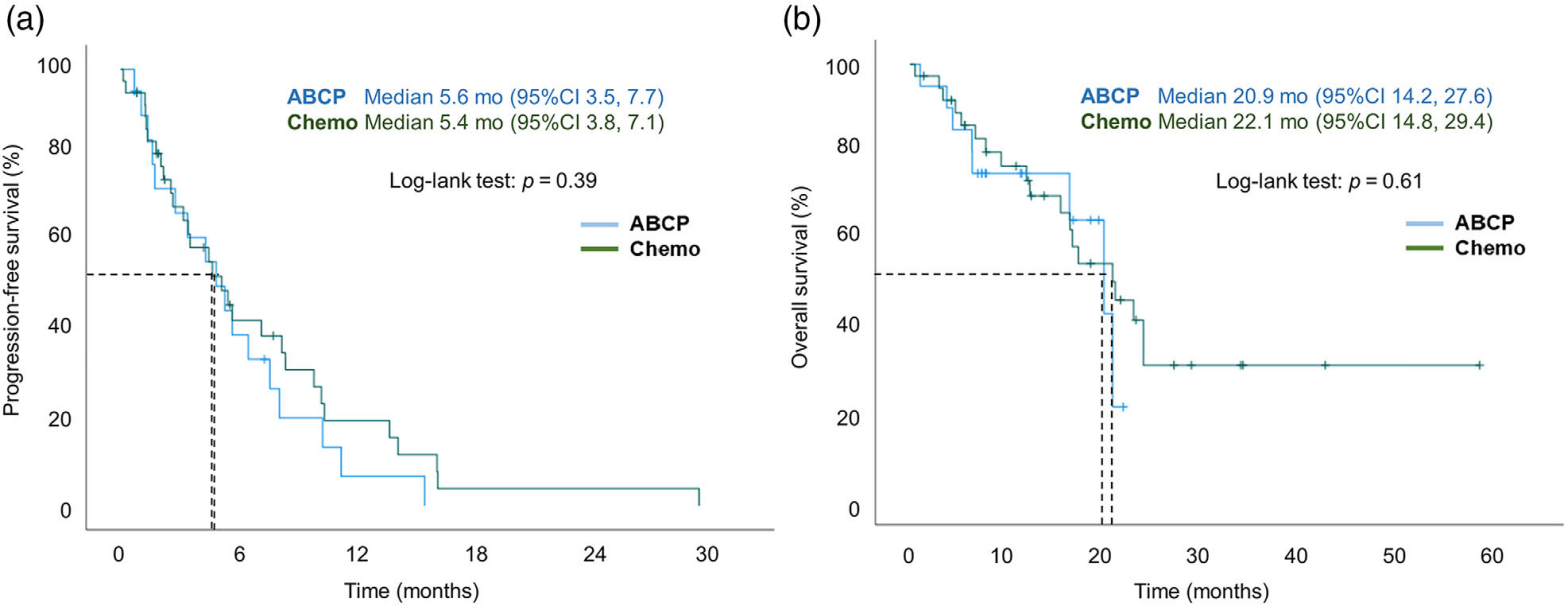
Kaplan–Meier analyses in the ABCP group versus the Chemo group in *EGFR*‐mutant patients: (a) PFS. (b) OS. ABCP, atezolizumab‐bevacizumab‐carboplatin‐paclitaxel; Chemo, chemotherapy; EGFR, epidermal growth factor receptor; OS, overall survival; PFS, progression‐free survival.

The median PFS were 5.6 and 5.4 months in the ABCP and Chemo groups, respectively (*p* = 0.39) (Figure 2(a)). The median OS were 20.9 and 22.1 months in the ABCP and Chemo groups, respectively (*p* = 0.61) (Figure 2(b)).

### Exploratory analysis of immunochemotherapy versus chemotherapy

**FIGURE 3:**
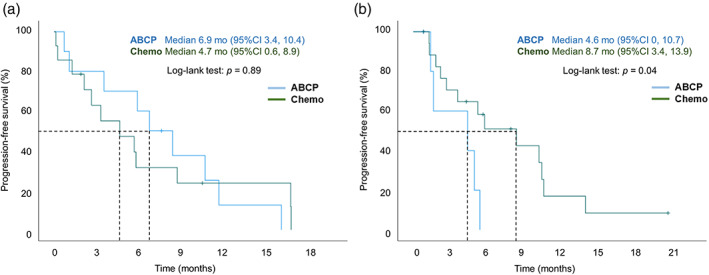
Kaplan–Meier analyses of PFS in the ABCP group versus the Chemo group according to PD‐L1 status in *EGFR*‐mutant patients: (a) PD‐L1–positive as ≥1%. (b) PD‐L1–negative as <1%. ABCP, atezolizumab‐bevacizumab‐carboplatin‐paclitaxel; Chemo, chemotherapy; EGFR, epidermal growth factor receptor, PD‐L1, programmed death‐ligand 1; PFS, progression‐free survival.

**FIGURE 4:**
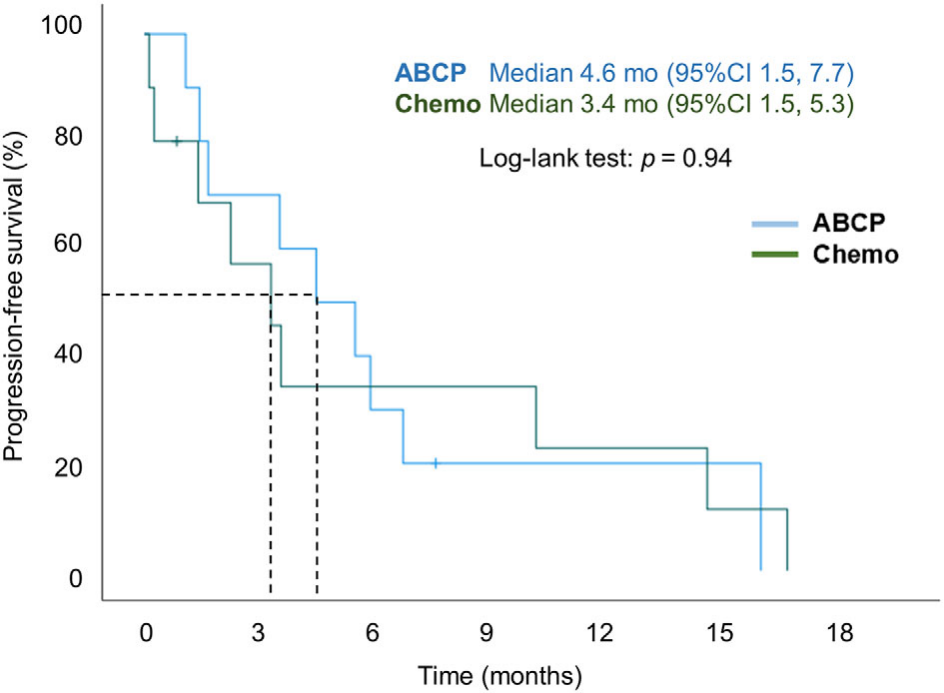
Kaplan–Meier analyses of PFS in the ABCP group versus the Chemo group in *EGFR*‐mutant patients with baseline brain metastases. ABCP, atezolizumab‐bevacizumab‐carboplatin‐paclitaxel; Chemo, chemotherapy; EGFR, epidermal growth factor receptor; PFS, progression‐free survival.

**FIGURE 5:**
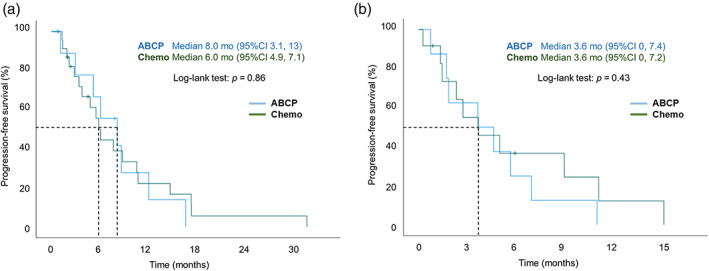
Kaplan–Meier analyses of PFS in the ABCP group versus the Chemo group according to *EGFR* mutation subgroup: (a) exon19 deletion. (b) exon21 L858R. ABCP, atezolizumab‐bevacizumab‐carboplatin‐paclitaxel; Chemo, chemotherapy; EGFR, epidermal growth factor receptor; PFS, progression‐free survival.

We further compared immunochemotherapy (*n* = 20) and platinum‐based chemotherapy (*n* = 37) to determine the subjects for whom each is effective. The results of the exploratory PFS analysis are shown in Figures 3, 4 and 5. In subgroup analyses according to the PD‐L1 status, limited PFS improvement was found in PD‐L1–positive patients in the ABCP group versus the Chemo group (6.9 months vs. 4.7 months, *p* = 0.89) (Figure 3(a)). In the PD‐L1–negative patients, the median PFS was significantly longer in the Chemo group than in the ABCP group (8.7 months vs. 4.6 months, *p* = 0.04) (Figure 3(b)). In addition, in the ABCP group, the median PFS was significantly longer in the PD‐L1–positive group than in the PD‐L1–negative group (*p* = 0.047).

The median PFS were 4.6 and 3.4 months in patients with baseline brain metastases in the ABCP and Chemo groups, respectively (*p* = 0.94) (Figure 4). For the sensitizing *EGFR* mutation, there was no difference in median PFS between the ABCP and Chemo group patients with exon 19 deletion (8.0 months vs. 6.0 months, *p* = 0.86) and exon 21 L858R (3.6 months vs. 3.6 months, *p* = 0.43). However, overall, the exon 19 deletion subgroup had a better median PFS than the exon 21 L858R subgroup (Figure 5).

### Efficacy according to chemotherapy regimen

There were no statistically significant differences in median PFS among the four chemotherapy groups (cisplatin‐based chemotherapy, with or without bevacizumab, carboplatin‐based chemotherapy, with or without bevacizumab) and the ABCP group, respectively (Figure S1).

Of the 37 patients who received platinum‐based chemotherapy, 33 received PEM and four received nab‐paclitaxel (nab‐PTX). The median PFS of chemotherapy including PEM and immunochemotherapy was similar (4.9 months vs. 5.1 months, *p* = 0.66), whereas median PFS of chemotherapy including nab‐PTX was longer than that of immunochemotherapy (8.7 months vs. 5.1 months, *p* = 0.24).

### Safety

Grade 3/4 treatment‐related adverse events (AEs) were reported in 12 patients (60%) of the ABCP group and in eight patients (21.6%) of the Chemo group. The most common Grade 3/4 AEs was neutropenia, which occurred in six patients (30%) in the ABCP group and 2 (5.4%) in the Chemo group. One of the ABCP group died from a brain hemorrhage during the treatment. Details of grade 3/4 treatment‐related AEs are provided in Table S2.

IrAEs were observed in six patients in the ABCP group (endocrine, two; skin, one; hepatic, one; hepatic and skin, one; pulmonary and skin, one). One patient in the ABCP group experienced grade 3 erythema, and other patients experienced grade 1 or 2 adverse events.

## DISCUSSION

Here, we investigated the optimal treatment following the use of EGFR‐TKIs for *EGFR*‐mutant patients in a real‐world setting. In our study, the efficacy of ABCP therapy and platinum‐doublet chemotherapy was similar in *EGFR‐*mutant patients. Of note, the median PFS of ABCP therapy was better than that of chemotherapy when the PD‐L1 expression was positive and significantly worse when the PD‐L1 expression was negative, even in *EGFR*‐mutated NSCLC patients. To the best of our knowledge, this is the first study to show that PD‐L1 expression is a determinant of treatment choice after TKI failure in *EGFR*‐mutant patients.

In previous clinical trials and studies, the efficacy of immunochemotherapy in *EGFR*‐mutant patients was inconsistent. A single‐arm phase 2 trial of toripalimab plus chemotherapy in China, all for patients after TKI treatment, showed the ORR of 50% and median PFS period of 6.5 months in 40 *EGFR*‐mutant patients.^11^ In previous single‐center retrospective studies, the median PFS and OS for the immunochemotherapy group were 4.23 and not reached in Shen et al.'s^13^ study and 6.4 and 12.8 months in Long et al.'s^14^ study, respectively. Zhang et al.^11^ were skeptical about treating all *EGFR*‐mutant patients with immunochemotherapy after TKI treatment. We have shown in a multicenter study that immunochemotherapy should not be applied to all *EGFR*‐mutant patients.

In this study, PD‐L1 expression was found to be an important factor in selecting immunochemotherapy in *EGFR*‐mutant patients. Previous studies have reported that PD‐L1 expression is associated with treatment response in ICI monotherapy for *EGFR*‐mutant patients. In the final analysis of the IMpower150 trial, which included all the patients, regardless of *EGFR* mutation, the median OS in PD‐L1–positive patients were 22.5 and 16 months in the ABCP and BCP groups, respectively. In contrast, for PD‐L1–negative patients, the median OS were 16.9 and 14.1 months in the ABCP and BCP groups, respectively.^15^ In the *EGFR* mutation group of the IMMUNOTARGET study, PD‐L1–negative patients had shorter median PFS than PD‐L1–positive patients (1.7 vs. 2.8 months, *p* = 0.01).^16^ Furthermore, Ichihara et al.^17^ reported that *EGFR*‐mutated patients with PD‐L1 ≥50% had significantly longer median PFS of immunotherapy alone compared to those with PD‐L1 <50% (6.4 vs. 1.4 months, *p* = 0.007). Masuda et al.^18^ also reported that PD‐1 antibody therapy showed better median PFS in *EGFR*‐mutant patients with high PD‐L1 expression than those with low PD‐L1 expression.

In our study, patients with brain metastases had longer median PFS with immunochemotherapy than with chemotherapy. Immunochemotherapy has been reported to be more promising than chemotherapy and combined chemotherapy and radiation therapy for patients with brain metastases.^19^ In another study, atezolizumab showed a clinical benefit over docetaxel for NSCLC patients with baseline brain metastases (median OS, 20.1 vs. 11.9 months).^20^ In addition, preclinical data showed increased CD4+ cells and resistance to Treg‐mediated suppression within the brain tumors in mice receiving immunotherapy.^21^ Immunochemotherapy may be a treatment option for *EGFR*‐mutant patients with brain metastases.

Regarding safety, grade 3/4 AEs in the ABCP group were reported in 66.7% of patients with *EGFR* mutations in the IMpower150 trial, which was comparable to the incidence in this study.^6^ Based on this study, particular attention should be paid to neutropenia when using ABCP therapy.

The present study has some limitations. First, the sample size of this study was small because ABCP therapy commenced at five participating institutions from 2019 or 2020, and EGFR‐TKI was prioritized for *EGFR*‐mutant patients. Because of the small sample size of this study, potential predictors may not have been found. In addition, several sub‐analyses had fewer than 10 patients. These issues may have biased the present findings and, therefore, the present results should be interpreted with caution. Second, a variety of regimens are included in the Chemo group. Therefore, we performed sub‐analyses comparing the treatment effect of each regimen to that of the ABCP group. The results showed no significant difference in median PFS, indicating that the analysis of the ABCP and Chemo groups was independent of the outcome of the specific chemotherapy regimen. Third, this study was retrospective in nature; therefore, information bias could not be excluded.

## CONCLUSIONS

In *EGFR*‐mutant patients, the efficacy of ABCP therapy was shown to be comparable to that of platinum‐based chemotherapy after EGFR‐TKI therapy. Notably, for PD‐L1–positive patients, ABCP therapy may be expected to have a better clinical outcome than chemotherapy; however, for PD‐L1–negative patients, platinum‐based chemotherapy may be recommended over ABCP therapy. The results of this study need to be further verified in the future.

## AUTHOR CONTRIBUTIONS

**Tae Hata**: conceptualization, data curation, investigation, writing, and editing. **Chikara Sakaguchi**: project administration, investigation, and writing – review. **Keita Hirano**: formal analysis and methodology. **Hiroshi Kobe**, **Masaki Ishida**, **Takayuki Nakano**, **Yusuke Tachibana**, **Nobuyo Tamiya**, **Shinsuke Shiotsu**, **Takayuki Takeda**, **Tadaaki Yamada**, **Toshihide Yokoyama**, and **Michiko Tsuchiya**: investigation. **Yukio Nagasaka**: supervision.

## CONFLICT OF INTEREST STATEMENT

Dr. Yamada reported honoraria from Eli Lilly Japan K.K. and research funding from ONO Pharmaceutical, Janssen Pharmaceutical K.K., CHUGAI Pharmaceutical, Takeda Pharmaceutical, and Pfizer. The other authors declare that they have no competing interests.

## Supporting information


**Figure S1.** Kaplan–Meier analyses of PFS in the ABCP group versus the Chemo group in *EGFR*‐mutant patients: (a) ABCP versus CDDP‐based chemotherapy including bevacizumab. (b) ABCP versus CDDP‐based chemotherapy not including bevacizumab. (c) ABCP versus CBDCA‐based chemotherapy including bevacizumab. (d) ABCP versus CBDCA‐based chemotherapy not including bevacizumab. ABCP, atezolizumab‐bevacizumab‐carboplatin‐paclitaxel; CBDCA, carboplatin; CDDP, cisplatin; Chemo, chemotherapy; EGFR, epidermal growth factor receptor; PFS, progression‐free survival.

**Table S1.** Details of platinum‐based chemotherapy.
**Table S2.** Treatment‐related adverse events (grade 3/4).

## Data Availability

The datasets used and/or analyzed during the current study are available from the corresponding author on reasonable request.
