# Supplementary figures and images for: Exploratory analysis of immunochemotherapy compared to chemotherapy after EGFR‐TKI in non–small cell lung cancer patients with EGFR mutation: A multicenter retrospective study
Source: Thorac Cancer. 2023 Mar 3;14(11):1004–11. doi: 10.1111/1759-7714.14836 (PMC10101833; doi:10.1111/1759-7714.14836)

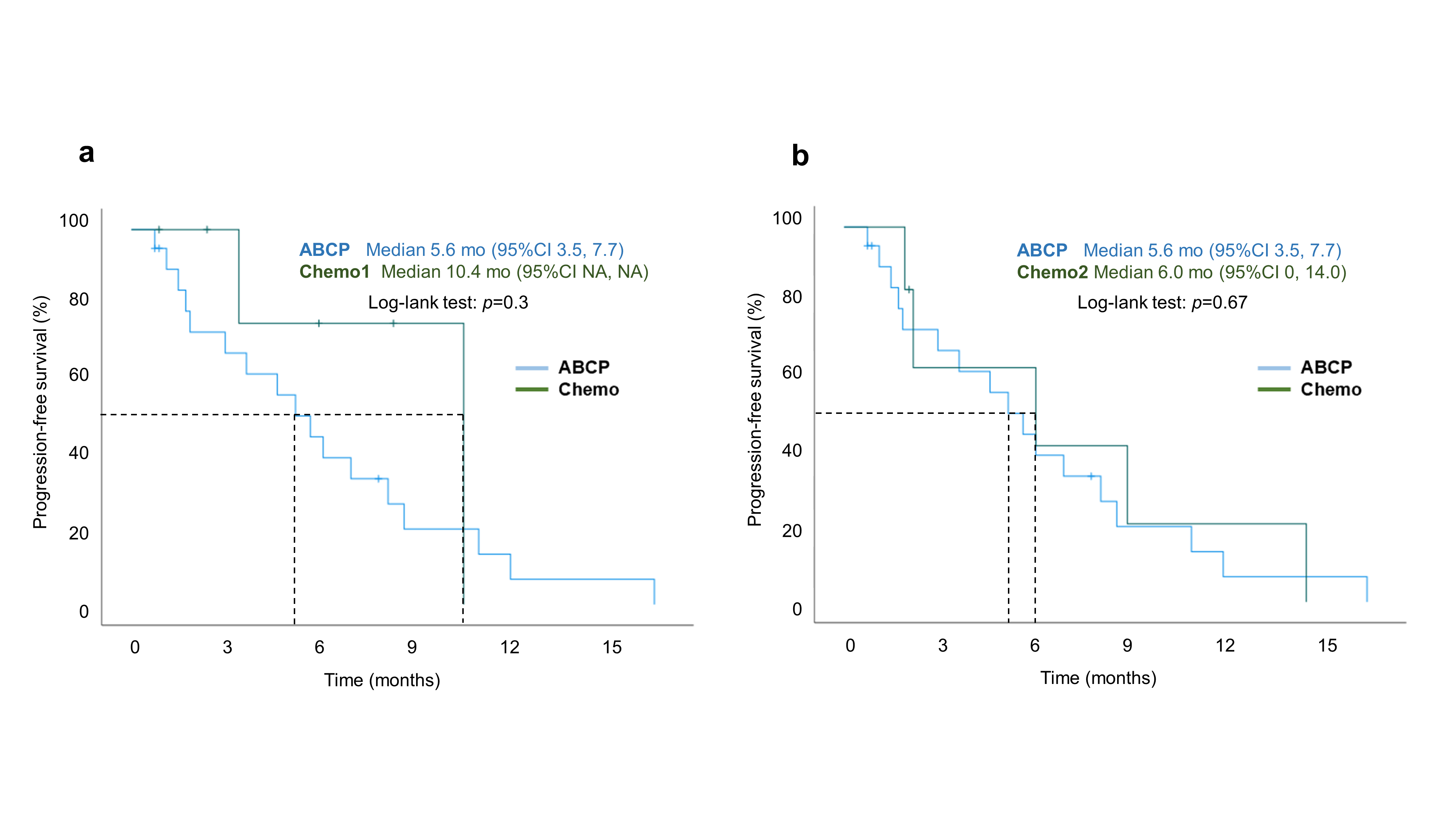

Supplement: Supplementary file 1 — Figure S1. Kaplan–Meier analyses of PFS in the ABCP group versus the Chemo group in EGFR‐mutant patients: (a) ABCP versus CDDP‐based chemotherapy including bevacizumab. (b) ABCP versus CDDP‐based chemotherapy not including bevacizumab. (c) ABCP versus CBDCA‐based chemotherapy including bevacizumab. (d) ABCP versus CBDCA‐based chemotherapy not including bevacizumab. ABCP, atezolizumab‐bevacizumab‐carboplatin‐paclitaxel; CBDCA, carboplatin; CDDP, cisplatin; Chemo, chemotherapy; EGFR, epidermal growth factor receptor; PFS, progression‐free survival. [file TCA-14-1004-s001.zip › tca14836-sup-0001-Figure S1ab.tif]

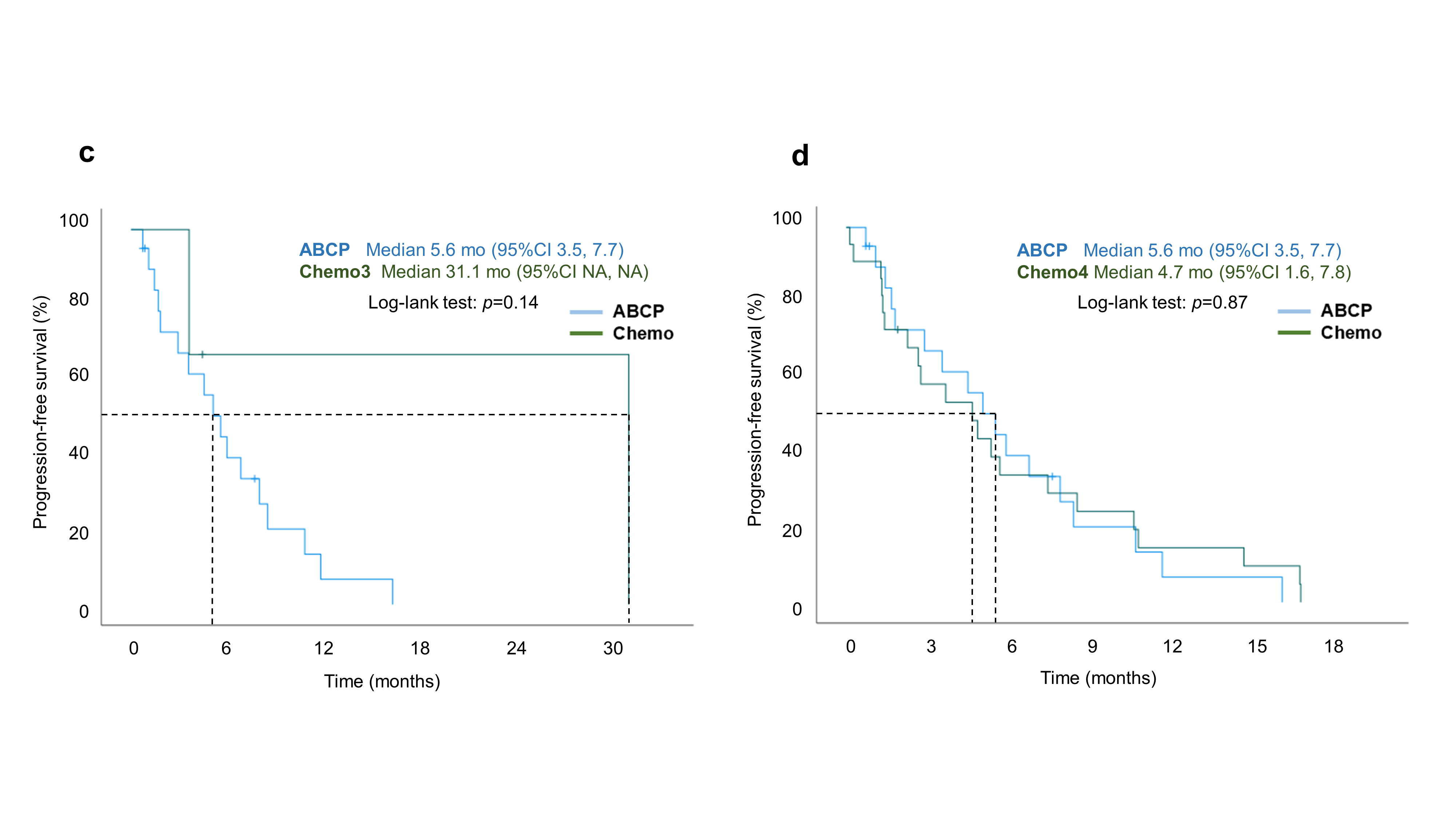

Supplement: Supplementary file 1 — Figure S1. Kaplan–Meier analyses of PFS in the ABCP group versus the Chemo group in EGFR‐mutant patients: (a) ABCP versus CDDP‐based chemotherapy including bevacizumab. (b) ABCP versus CDDP‐based chemotherapy not including bevacizumab. (c) ABCP versus CBDCA‐based chemotherapy including bevacizumab. (d) ABCP versus CBDCA‐based chemotherapy not including bevacizumab. ABCP, atezolizumab‐bevacizumab‐carboplatin‐paclitaxel; CBDCA, carboplatin; CDDP, cisplatin; Chemo, chemotherapy; EGFR, epidermal growth factor receptor; PFS, progression‐free survival. [file TCA-14-1004-s001.zip › tca14836-sup-0002-Figure S1cd.tif]
