# Supplementary material for: Exploratory analysis of immunochemotherapy compared to chemotherapy after EGFR‐TKI in non–small cell lung cancer patients with EGFR mutation: A multicenter retrospective study
Source: Thorac Cancer. 2023 Mar 3;14(11):1004–11. doi: 10.1111/1759-7714.14836 (PMC10101833; doi:10.1111/1759-7714.14836)
Supplement: Supplementary file 2 — Table S1. Details of platinum‐based chemotherapy. Table S2. Treatment‐related adverse events (grade 3/4). [file TCA-14-1004-s002.docx]

**Supplementary Table 1.** Details of platinum-based chemotherapy

| Treatment (*n* = 37) | 2nd line  (*n* = 33)  No (%) | 3rd line  (*n* = 4)  No (%) | Total  (*n* = 37)  No (%) |
| --- | --- | --- | --- |
| CDDP + chemotherapy* (with BEV) | 4 (10.8) | 2 (5.4) | 6 (16.2) |
| CDDP + chemotherapy (without BEV) | 6 (16.2) | 0 | 6 (16.2) |
| CBDCA + chemotherapy (with BEV) | 2 (5.4) | 1 (2.7) | 3 (8.1) |
| CBDCA + chemotherapy (without BEV) | 21 (56.8) | 1 (2.7) | 22 (59.5) |

* chemotherapy regimen including pemetrexed (*n* = 33) and nab-paclitaxel (*n* = 4)

*BEV* bevacizumab, *CBDCA* carboplatin, *CDDP* cisplatin

**Supplementary Table 2.** Treatment-related adverse events (grade3/4)

|  | **ABCP (n = 20)**  **No (%)** | | **Chemo (n = 37)**  **No (%)** | | **All (n = 57)**  **No (%)** | |
| --- | --- | --- | --- | --- | --- | --- |
|  | Grade 3 | Grade 4 | Grade 3 | Grade 4 | Grade 3 | Grade 4 |
| Neutrophile count decreased | 1 (5) | 5 (25) | 2 (5.4) | 0 | 3 (5.3) | 5 (8.8) |
| Peripheral neuropathy | 1 (5) | 0 | 0 | 0 | 1 (1.8) | 0 |
| Erythema | 1 (5) | 0 | 0 | 0 | 1 (1.8) | 0 |
| Hypertension | 1 (5) | 0 | 0 | 0 | 1 (1.8) | 0 |
| Alanine aminotransferase increased/Aspartate aminotransferase increased | 1 (5) | 0 | 0 | 0 | 1 (1.8) | 0 |
| Proteinuria | 1 (5) | 0 | 0 | 0 | 1 (1.8) | 1 (1.8) |
| Heart failure | 1 (5) | 0 | 0 | 0 | 1 (1.8) | 0 |
| Hemophagocytic syndrome | 1 (5) | 0 | 0 | 0 | 1 (1.8) | 0 |
| Febrile neutropenia | 0 | 1 (5) | 0 | 0 | 0 | 1 (1.8) |
| Pancreatitis | 0 | 0 | 2 (5.4) | 0 | 2 (3.5) | 0 |
| Pneumonitis | 0 | 0 | 1 (2.7) | 0 | 1 (1.8) | 0 |
| Mucositis oral | 0 | 0 | 1 (2.7) | 0 | 1 (1.8) | 0 |
| Colonic perforation | 0 | 0 | 1 (2.7) | 0 | 1 (1.8) | 0 |
| Pulmonary embolism | 0 | 0 | 1 (2.7) | 0 | 1 (1.8) | 0 |
| Platelet count decreased | 0 | 0 | 0 | 1 (2.7) | 0 | 1 (1.8) |

※grade 5 cerebral hemorrhage in the ABCP group (n=1)

**Supplementary Figure legends**

**Supplementary Figure 1.** Kaplan–Meier analyses of PFS in the ABCP group versus the Chemo group in *EGFR*-mutant patients: (a) ABCP versus CDDP-based chemotherapy including bevacizumab. (b) ABCP versus CDDP-based chemotherapy not including bevacizumab. (c) ABCP versus CBDCA-based chemotherapy including bevacizumab. (d) ABCP versus CBDCA-based chemotherapy not including bevacizumab. (*ABCP* atezolizumab-bevacizumab-carboplatin-paclitaxel, *CBDCA* carboplatin, *CDDP* cisplatin, *Chemo* chemotherapy, *EGFR* epidermal growth factor receptor, *PFS* progression-free survival)
